# Chitosan Nanoparticles as Potential Nano-Sorbent for Removal of Toxic Environmental Pollutants

**DOI:** 10.3390/nano13030447

**Published:** 2023-01-21

**Authors:** Asmaa Benettayeb, Fatima Zohra Seihoub, Preeti Pal, Soumya Ghosh, Muhammad Usman, Chin Hua Chia, Muhammad Usman, Mika Sillanpää

**Affiliations:** 1 Laboratoire de Génie Chimique et Catalyse Hétérogène, Département de Génie Chimique, Université de Sciences et de la Technologie-Mohamed Boudiaf, USTO-MB, BP 1505 EL-M’NAOUAR, Oran 31000, Algeria; 2 Accelerated Cleaning Systems India Private Limited, Sundervan Complex, Andheri West, Mumbai 400053, India; 3 Department of Genetics, Faculty of Natural and Agricultural Sciences, University of the Free State, Bloemfontein 9301, South Africa; 4 School of Civil Engineering, Hamburg University of Technology, Am Schwarzenberg-Campus 3, 20173 Hamburg, Germany; 5 Department of Applied Physics, Faculty of Science and Technology, Universiti Kebangsaan Malaysia, Bangi 43600, Selangor, Malaysia; 6 PEIE Research Chair for the Development of Industrial Estates and Free Zones, Center for Environmental Studies and Research, Sultan Qaboos University, Al-Khoud, Muscat 123, Oman; 7 Department of Chemical Engineering, School of Mining, Metallurgy and Chemical Engineering, University of Johannesburg, Doornfontein 2028, South Africa; 8 School of Chemical and Metallurgical Engineering, University of the Witwatersrand, Johannesburg 2050, South Africa; 9 Chemistry Department, College of Science, King Saud University, Riyadh 11451, Saudi Arabia; 10 School of Resources and Environment, University of Electronic Science and Technology of China (UESTC), No. 2006, Xiyuan Ave., West High-Tech Zone, Chengdu 611731, China; 11 Faculty of Science and Technology, School of Applied Physics, University Kebangsaan Malaysia, Bangi 43600, Selangor, Malaysia

**Keywords:** nano-sorbent, adsorption, chitosan nanoparticles, pollutant and metal recovery, functionally flexible adsorbent, water treatment

## Abstract

Adsorption is the most widely used technique for advanced wastewater treatment. The preparation and application of natural renewable and environmentally friendly materials makes this process easier and more profitable. Chitosan is often used as an effective biomaterial in the adsorption world because of its numerous functional applications. Chitosan is one of the most suitable and functionally flexible adsorbents because it contains hydroxyl (-OH) and amine (-NH_2_) groups. The adsorption capacity and selectivity of chitosan can be further improved by introducing additional functions into its basic structure. Owing to its unique surface properties and adsorption ability of chitosan, the development and application of chitosan nanomaterials has gained significant attention. Here, recent research on chitosan nanoparticles is critically reviewed by comparing various methods for their synthesis with particular emphasis on the role of experimental conditions, limitations, and applications in water and wastewater treatment. The recovery of pollutants using magnetic nanoparticles is an important treatment process that has contributed to additional development and sustainable growth. The application of such nanoparticles in the recovery metals, which demonstrates a “close loop technology” in the current scenarios, is also presented in this review.

## 1. Introduction

The diversity and complexity of pollutants greatly affect the efficiency of wastewater treatment [1]. To overcome this limitation, extensive research has focused on the development of biosorbents [2,3] and their variable applications with the help of nanotechnology [3,4]. Nanotechnology exploits the properties of any material at the nanoscale. The materials of this new technology are termed nanoparticles (NPs) [5]. Nanotechnology is an ideal solution to ensure high quality water. It can be considered a powerful 21st century tool for protecting the environment and improving environmental quality [6]. The application of nanotechnology in water purification and environmental sanitation has potential, as conventional methods do not always provide cost-effective solutions for the removal of common pollutants. Conventional technologies have a limited lifetime, generate hazardous and toxic environmental wastes, and are non-renewable. For several years, NPs have been the subject of numerous research publications, and patents, because of their large surface area, high resistance to heat and chemicals, and high adsorption capacity for the removal of organic and inorganic contaminants [7,8,9,10,11].

Adsorption technology is one of the most reliable strategies in wastewater treatment, and the use of a variety of nanosized adsorbents enables preferential surface adsorption [12,13,14,15,16]. The increase in surface area can increase the sorption capacity towards pollutants on the surface of NPs [6,17,18,19,20]. In addition to wastewater treatment, NPs are used as antimicrobial agents [21], as catalysts [22], in biomedicine, energy conversion [23], agriculture, electronics, and optoelectronics industries [24]. According to Vakili et al. (2014), nano-chitosan is one such nanomaterial that is a natural substance with excellent physicochemical properties and is harmless to humans [25]. Therefore, chitosan biopolymer has become the environmentally friendly substance of choice. Several modifications have been carried out on the alginate for the introduction of the amine functional group (-NH_2_) on its surface [26,27,28], or other biosorbents by introducing other actives functions [29,30,31,32,33,34], this active site is found naturally in chitosan. Chitosan is rich in amino (-NH_2_) and hydroxyl (-OH) groups, which gives it a powerful adsorption capacity and reactivity to most pollutants [35,36,37,38]. Thus, chitosan is an excellent natural adsorbent that can be modified to increase its efficiency and improve its basic properties [6,17,39,40,41].

The main problem with using chitosan in its natural form is its low adsorption capacity, which can be improved by physical or chemical modification. Hence, researchers have developed more effective chitosan-based adsorbents. Chitosan NPs are among the best nano-adsorbents due to their large surface area, high adsorption capacity, and environmental friendliness. Chitosan is abundant in nature, reusable, and can be easily modified with various chemical and biological agents so that it can be regenerated and reused over several cycles. Chitosan NPs can be categorised as nano adsorbents that meet the essential criteria for use in wastewater treatment. Chitosan NPs can be chemically inert, and their morphology resists various complex conditions.

The preparation of chitosan NPs cannot only improve the surface area and adsorption capacity, but the presence of functional groups also makes it selective [42,43,44]. Since chitosan is biodegradable, it does not cause additional environmental pollution. Apart from its ecological nature, it also has antibacterial properties that enhance its use as an adsorbent for water treatment. According to Saxena et al. (2020), it needs chemical modification using chemical cross-linking to increase its stability over time [45].

In the present review, the basic properties of chitosan NPs and chitosan magnetic NPs, including chitosan nanocomposites, and other types based on chitosan NPs are discussed. Some effective preparation methods, such as covalent crosslinking, ionic gelation, change in pH, and other methods are also discussed. The characteristics of chitosan and operational conditions are compared in terms of their efficiency as adsorbents for the removal of various pollutants, such as uranium, and rare metals. The parameters affecting the adsorption mechanism, and advantages and limitations of using chitosan NPs in fields of sorption/biosorption are presented.

## 2. Unique Properties of Chitosan Nanomaterials and Magnetic Chitosan

Chitosan (poly [β-(1-4)-2-amino-2-deoxy-D-glucopyranose]) is a linear cationic biopolymer with high molecular weight. The primary source of usable chitosan is the deacetylation of chitin obtained from the shells of crustaceans (crabs, lobsters, shrimp, and crayfish) [46], which are an abundant natural resource. The natural material is commercially obtained from the deacetylation of chitin by thermochemical treatment [35]. Natural chitosan is non-toxic, non-allergenic, biodegradable, biocompatible, inexpensive, hydrophilic, biologically active, and can form fibers and films [47,48,49,50].

**Figure 1:**
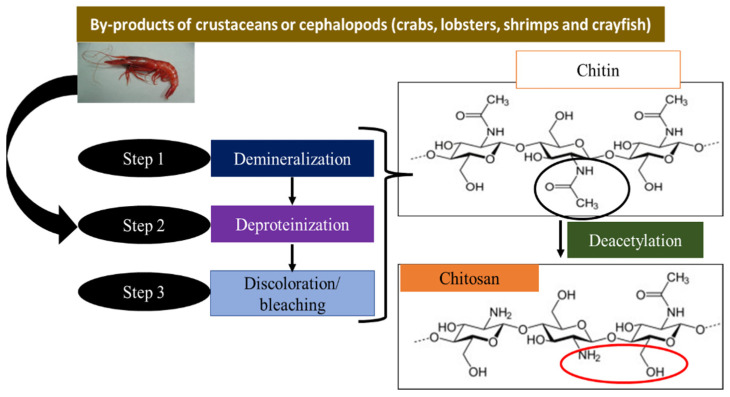
Different stages of the chitosan manufacturing process.

Figure 1 summarizes the chemical process to obtain chitosan from chitin. Chitosan is a semi-crystalline cationic polysaccharide that attracts positively charged molecules and enhances bonding due to the presence of the –NH_2_ group. In addition, the –OH group is also present in the structure and helps to increase the adsorption capacity [51].

Nano-sized particles have characteristics that cannot be achieved with solid, normal-sized material. For example, the electronic and optical properties of metallic materials can be modified by controlling their size below the Bohr radius (usually between 1–10 nm). The interest in NPs is due to their ability to act as an effective bridge between solid materials and atomic structures. Solid materials exhibit constant physical properties, regardless of their size and mass. However, NPs have properties that depend on their size due to the large proportion of atoms on their surfaces relative to their volume, resulting in a large specific surface area. In view of this, the electronic, optical, and magnetic properties of materials change as their size decreases towards the nanoscale. Therefore, controlling the size of NPs is of particular interest because it can influence their properties. The exceptional physicochemical properties of nanomaterials are due to three main reasons:

(i) The size of the nanomaterial is comparable to the Bohr radius of the excitons. This dramatically alters the optical, luminescent, and redox properties of nanomaterials compared to their bulk counterparts.
(ii) The surface area atom largely determined the thermodynamic properties of solids and also determines the melting temperature and structural transitions of nanomaterials.
(iii) When the particle size is decreased, the net internal cohesive forces increase. Thus, reducing the particle size increases the surface area to bulk volume ratio, i.e., particle size is inversely proportional to the surface area to bulk volume ratio [52].

In general, nanotechnology is used to produce materials with specific properties and a high degree of reproducibility. In this regard, researchers are currently focusing on the synthesis of new nanostructured materials capable of cleaning the environment; they know that nanomaterials are entirely or partially composed of nano-objects, which gives them improved or specific properties in nanometric dimensions. In the family of nanomaterials, there are three types, namely NPs, nano-fibers, and nano-films. NPs are elements with a nanometric size between 1–100 nm [53] and are used daily in products such as cosmetics, paints, electronics, and computers. NPs can be in the form of powders, suspensions, solutions, or gels from which other physical forms, such as nano-beads are formed.

In recent years, researchers have focused on the use of NPs, particularly magnetic NPs, as effective adsorbents for the treatment of pollutants present in wastewater [54,55].

Magnetic NPs have been used as adsorbents for water treatment. These adsorbents have remarkable properties such as nanometric size, high specific surface area per volume ratio, and resistance to internal diffusion leading to a high adsorption capacity [56,57], biocompatibility, biodegradability and low toxicity [58], low cost of fabrication, green sources, magnetic intensity, so, for these reasons that chitosan magnetic received considerable attention.

**Figure 2:**
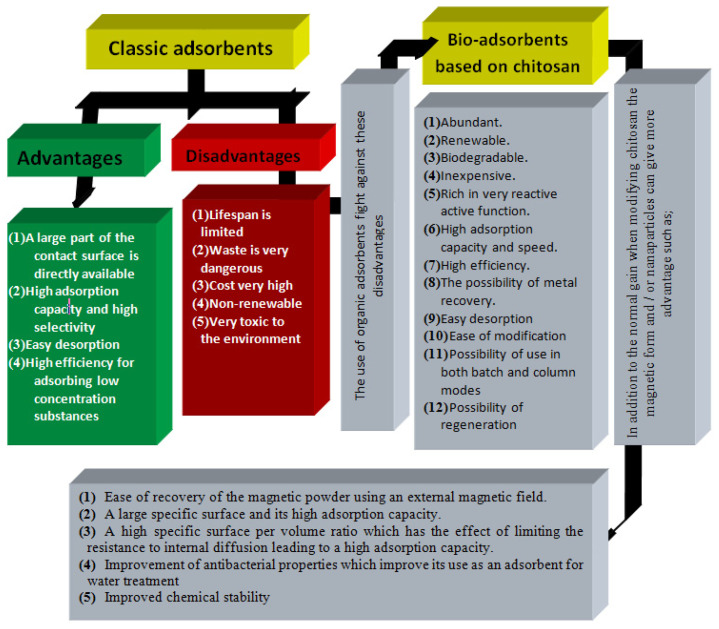
Advantages and disadvantages of NPs and use of chitosan NPs to overcome the disadvantages of traditional NPs in wastewater treatment.

In addition, iron oxide NPs have the advantage of being superpara-magnetic [59] and the powder can be easily recovered using an external magnetic field. Magnetic separation technology is easy to use and preferable to avoid slow separation techniques such as filtration, centrifugation, and precipitation. Some important characteristics of nano-adsorbents compared to classical ones are summarized in Figure 2.

## 3. Standard Methods of Chitosan Nanoparticles Synthesis

### 3.1. Synthesis of Chitosan NPs

**Figure 3:**
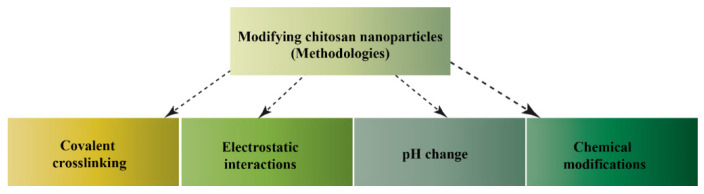
Methods to modify chitosan NPs.

Research shows that chitin and chitosan can be easily transformed into gels, membranes, nanofibers, beads, and micro-nanoparticles. Generally, there are four ways of modifying chitosan to obtain NPs, which are summarized in Figure 3.

#### 3.1.1. Covalent Cross-Linking Methodologies

Different cross-linking agents can be used for the fabrication of chitosan cross-linking especially glutaraldehyde because it can be easily used in various physicochemical reactions to prepare functional materials from amino polysaccharides, proteins, natural and synthetic polymers due to the -NH_2_ and -OH groups. Ohya et al. (1994) obtained chitosan NPs with a diameter between 250 and 300 nm by emulsifying the chitosan solution in toluene and then using glutaraldehyde for covalent cross-linking [60]. Banerjee et al. (2002) obtained chitosan NPs with a reverse micelle method using a surfactant (sodium bis (ethylhexyl) sulfosuccinate) in hexane. The aqueous core of the micelles contained a chitosan solution, which was cross-linked with glutaraldehyde [61]. The researchers confirmed that the particle size depended on the rate of cross-linking with glutaraldehyde. The obtained NPs between 30 to 100 nm when cross-linked with 10% and 100% of the amine, respectively [61].

#### 3.1.2. Ionic Gelation by Electrostatic Interactions, Encapsulation, or Adsorption

**Figure 4:**
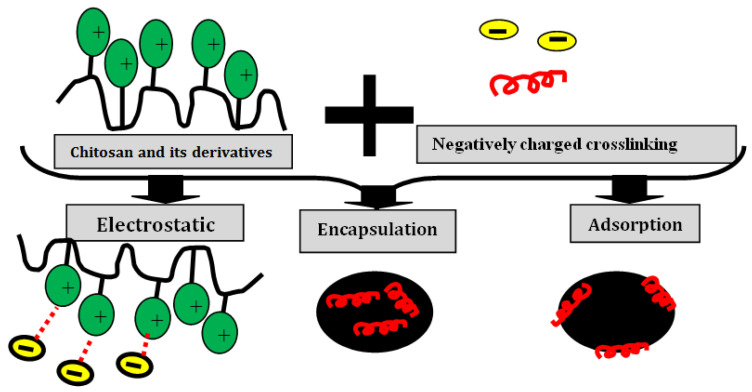
Schematic representation of different techniques for obtaining NPs based on chitosan and a cross-linking agent carrying an opposite charge.

The ionic gelation for chitosan nanoparticle synthesis with the cross-linking of tripolyphosphate is another method that is widely method due to its simplicity and ease of preparation [62,63]. Synthesis of chitosan NPs uses cross-linking agents with opposite charges, causing an electrostatic attraction, encapsulation, or adsorption has also been applied (Figure 4).

These researchers [62,63] confirmed the formation of NPs of different sizes by adding a tripolyphosphate solution to an acidic chitosan solution. They know that other anionic spaces can play the same role as tripolyphosphate.

In the work of Ali et al. (2018), chitosan NPs were prepared according to the method of Qi and Xu, (2004) by dissolving 0.5 g of chitosan in 100 mL of acetic acid (1% (*v*/*v*)) and adjusting the pH of the solution to a 4.6–4.8 using 1N NaOH. Chitosan NPs formed spontaneously after adding 3 mL of chitosan solution under vigorous magnetic stirring to 1.0 mL of an aqueous solution of sodium tripolyphosphate (0.25% *w*/*v*), with a chitosan/tripolyphosphate ratio of 3/1 at room temperature [64,65]. In this type of modification, the formation of chitosan NPs is attributed to the aid of electrostatic attraction between the positively charged amine group of chitosan and the anionic cross-linking agent such as tripolyphosphate. The synthesis can be carried out by adding polyethylene glycol as a cross-linking agent to the solution of chitosan in the acetic acid-containing stabilizer. The size and surface charge of NPs depend on the ratio of chitosan to the stabilizer. Generally, the size of the chitosan NPs obtained by this method ranges from 40–100 nm [64,66].

#### 3.1.3. Change in pH

The microemulsion method is one of the newer techniques for preparing inorganic NPs. Microemulsions are isotropic, macroscopically homogeneous, and thermodynamically stable solutions containing at least three components, a polar phase (usually water), a non-polar phase (usually oil), and a surfactant [67].

Brunel et al. (2008) developed a technique to obtain chitosan nanogels without chemical cross-linking. The principle is to apply an aqueous gelation process to a water/oil reverse microemulsion. In this method of manufacturing chitosan nanogels, an acid solution of chitosan is dispersed in an organic phase containing a surfactant and then the nanodroplets are gelled by modifying the pH of the medium [68]. The chitosan nano-hydrogels are then washed to remove the organic phase and redispersed in an acid buffer. During emulsification, the product formation depends on the diffusion rate of the solvent in the dispersed phase. The ratio of stabilizer and oil-polymer in an aqueous solution offers greater solvent diffusion into the external phase [69].

#### 3.1.4. Chemical Modification of Chitosan

Chemical modification of chitosan with hydrophobic groups is used to obtain an amphiphilic polymer with self-assembly properties in solution. The grafting of these groups occurs generally via the free amine functions of chitosan. Chitosan, which has become amphiphilic, then organizes into sub-micronic core-shell structures in an aqueous medium. The hydrophobic segments are located in the core of the particle, while the hydrophilic segments form the crown. The size of the NPs depends on the grafted molecule and the rate of substitution [70,71]. Furthermore, according to Morales et al. (2013) [72], the size of NPs increases with increasing the amount of cross-linking agents. For example, in the work of Galhoum et al. (2015), the size of the crystallites of magnetic particles varies between 11 and 13 nm when cross-linked with glutaraldehyde [73]. When tripolyphosphate is used as a crosslinking agent with a chitosan/TPP ratio of (3:1), we can obtain chitosan NPs of small size [65].

### 3.2. Synthesis of Chitosan-Magnetic NPs

**Figure 5:**
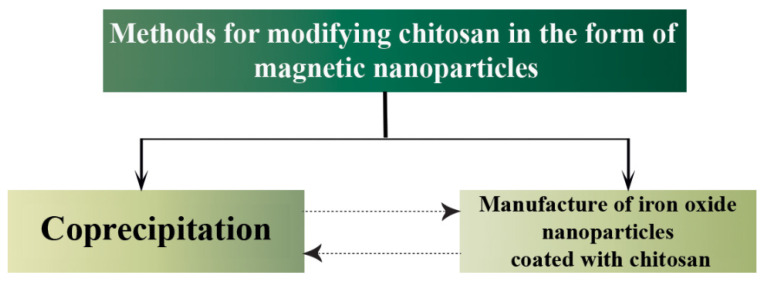
A well-known method of modifying chitosan to form magnetic NPs.

Several methods have been used to prepare high-quality magnetic NPs of different sizes. Co-precipitation (Figure 5), thermal decomposition, hydrothermal and solvothermal methods, microemulsion, and sol-gel methods are used for the preparation of magnetic chitosan NPs. The most commonly used manufacturing methods are co-precipitation and microemulsion. The ease of preparation of chitosan and the fact that NPs intermix during co-precipitation is an important advantage in the use of magnetic composites. The functional groups on the surface and iron oxide react easily with chitosan and its derivatives [74]. Moreover, there is a link between co-precipitation and iron oxide-chitosan blends, so we can obtain magnetic NPs when we use either method, but, in the case of chitosan, we need to merge both two methods to obtain magnetic NPs from chitosan.

From several methods of manufacturing magnetic chitosan, we can conclude that magnetic chitosan can be prepared in a one-step or two-step process. In the one-step process, ferric and ferrous salts (Fe_3_O_4_) or (γ-Fe_2_O_3_) are dissolved in a chitosan solution by raising the pH of the solution (above 9). Then, magnetite formation and magnetite/chitosan precipitation take place simultaneously in one pot [75,76]. In the two-step method, the magnetic particles and the chitosan solution are prepared separately, then the magnetic particles are dispersed in the chitosan solution, followed by a precipitation step and crosslinking to finally form the magnetic chitosan.

#### 3.2.1. Chemical Co-Precipitation

Chemical co-precipitation of Fe(II) and Fe(III) in alkaline solution (changing pH) is widely used for the preparation of magnetite (Fe_3_O_4_) or (γ-Fe_2_O_3_) NPs due to its simplicity, reproducibility, energy efficiency, and the possibility of large-scale preparation.

This technique has been used by several researchers such as Namdeo and Bajpai (2008) and Gregorio-Jauregui et al. (2012) [35,77]. NPs were prepared with sizes ranging from 5 to 100 nm using co-precipitation. Chemical co-precipitation is based on the reaction of aqueous solutions of Fe(II)/Fe(III) salts, usually in a 1/2 molar ratio with a base such as ammonia, potassium hydroxide, or sodium hydroxide under an inert atmosphere at 40–50 °C. The size and shape of the magnetite particles are generally controlled by the synthesis conditions such as temperature, pH, ionic strength, Fe(II) and Fe(III) concentrations, and the nature and concentration of the base.

#### 3.2.2. Iron Oxide-Chitosan Blends

Most of the literature dealing with magnetic NPs for the decontamination of wastewater by removing heavy metals relates to magnetic NPs encapsulated in polymers, especially chitosan. The chitosan biopolymer is grafted onto the surface of the magnetic cores, or the magnetic powder is encapsulated in chitosan using different syntheses to prepare Fe_3_O_4_ NPs modified by chitosan (magnetic core and multi-core in chitosan-based adsorption material [78]). Several methods, such as co-precipitation [79], cross-linking [80], and covalent bonding using coupling agents can be used to prepare new materials. The resulting magnetic composites are mostly micrometric in size, have low stability, and aggregate.

There are several ways to manufacture magnetic chitosan NPs; the preferred and simplest method is proposed by Galhoum et al. (2015a) and Galhoum et al. (2015c). The method consists of the synthesis of magnetic NPs with the deposition of a biopolymer on magnetic cores. Magnetic NPs are synthesized by a hydrothermal process in a single vessel involving co-precipitation under thermal conditions with Fe(III) and Fe(II) salts in the presence of chitosan. The magnetic/chitosan composite material is cross-linked with epichlorohydrin and modified by the grafting/functionalization of amino acids such as alanine, cysteine, and serine [73,81,82,83,84]. The crystal size of magnetic NPs in the work of [85] has been found to be close to 13.5 nm, while in [81], the nanometric size of diethylenetriamine-functionalized chitosan magnetic nano-based particles is in the range of 30–50 nm.

According to Liu et al. (2015), magnetic chitosan nanoparticles (MCNPs) with a diameter of about 10 nm, can be prepared by co-precipitation in the following steps. Dissolve 0.5 g of chitosan in 200 mL of acetic acid (0.5% *v*/*v*) with continuous stirring. Add 4.7 g ${\mathrm{FeCl}}_{3}$·$H_{2}O\;$and 2.4 g ${\mathrm{FeSO}}_{4}$·$7H_{2}O$, which was dissolved in 22 mL of distilled water. Mix with the chitosan solution for 20 min at a stable stirring speed of 1000 rpm. Add dropwise 40 mL of ammonia (NH_4_OH) to the reaction system and 6 mL during continuous stirring at 1000 rpm for 3 h. Finally, the resulting magnetic chitosan NPs are separated by a magnetic field [85].

A second preparation method is reported by Zhou et al. (2014) where chitosan and magnetic NPs are bound to glutaraldehyde using cyclohexane, 10 mg/mL chitosan, 2% acetic acid, and Fe_3_O_4_ in water. The materials are mixed in a beaker at 1800 rpm. After that, 20 mL NaOH at a concentration of 50 mol/L is added quickly to the solution, and the mixture is placed in a water bath at 60 °C for 2 h [86].

**Figure 6:**
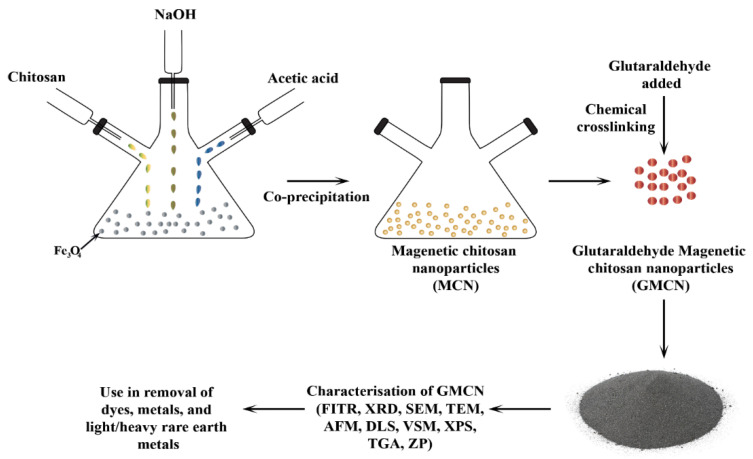
Reaction method of magnetic chitosan NPs cross-linked by glutaraldehyde.

**Figure 7:**
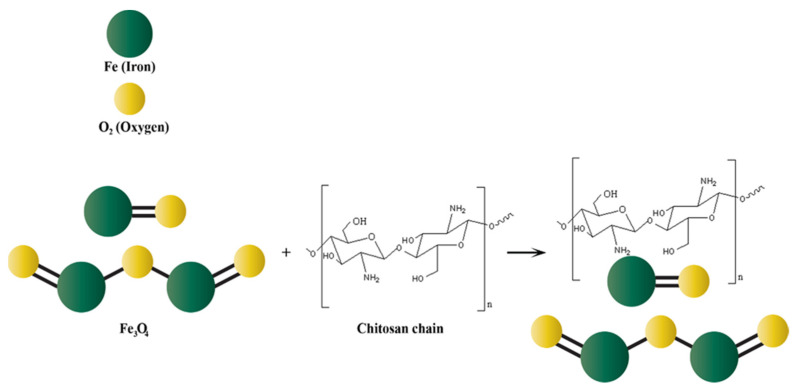
Interaction between the chitosan chain and the magnetic particles.

Chitosan magnetic NPs are collected with a magnet and rinsed with ethanol, then washed with deionized water three times. Finally, the NPs are modified by adding different concentrations of glutaraldehyde with constant stirring at 150 rpm for 60 min at room temperature to obtain chitosan magnetic NPs. A diagrammatic representation of the process is shown in Figure 6, and the interaction between the chitosan chain and magnetic particles Fe_3_O_4_ is shown in Figure 7. Ionic liquids also play an important role in the improvement of chitosan derivatives [87,88,89].

## 4. Characteristics of Chitosan NPs and Their Applications

According to Dasgupta et al. (2015), the physicochemical properties of NPs depend on their surface characteristics. Unlike chemical compounds, where the characterization is usually confined to chemical composition and purity, nanomaterials demand comprehensive characterization. Therefore, an extensive and complete characterization, including size distribution, shape, surface area, surface chemistry, crystallinity, porosity, agglomeration state, surface charge, and solubility is recommended for nanomaterials [90]. There are several methods of characterization to confirm the formation of NPs and to examine the efficiency of the nanomaterials with the target pollutant. The following table (Table 1) summarizes some types of analyzes (FTIR/SEM/TEM/XRD etc.) and their respective functions for the analysis of chitosan NPs.

### 4.1. Application of Chitosan NPs in Wastewater Treatment

Different protocols can be involved during the manufacture of chitosan magnetic NPs to produce, for example, chitosan-magnetite nanocomposites. These are synthesized by chemical co-precipitation of Fe(II) and Fe(III) ions by NaOH in the presence of chitosan and have been evaluated for the adsorption of several pollutants. Chitosan magnetic NP-based adsorbents adsorb differently and the best performance is achieved during grafting functional groups such as amino, sulfur, carboxyl, and alkyl groups onto the backbone of chitosan. Therefore, there are several modifications that increase the percentage of active sites (–NH_2_, –COOH, –SH, and –OH) responsible for the fixation of heavy metals, which consequently increase adsorption/sorption efficiency, different types of interaction between chitosan NPs and pollutants such as ion exchange, electrostatic attraction, and others according to the results of isotherms and kinetics, all this information gives us a clear idea about the mechanisms.

New chitosan magnetic NPs combine the metal/dye-binding potential of chitosan with an enormous surface area, dispersed character, and easy regeneration when a magnetic field of suitable strength is applied. Chitosan NPs exhibit good adsorption for dyes and heavy metal ions. It adsorbs radioactive heavy metals and rare earth metals effectively, with additional modifications improving adsorption capacity and efficiency.

#### 4.1.1. Heavy Metal Removal

The term heavy metal defines any metallic element with an atomic weight between 63.5 and 200.6 and a density greater than 5.0 [91]. Current research suggests that chitosan NPs with large specific surface areas and the inclusion of –NH_2_ and –OH groups, could effectively remove metal ions [17]. In addition, in the adsorption process, the magnetic separation advancements are effective, fast, and cost-effective compared to other separation methods since magnetic chitosan can be easily removed using an external magnetic field from the media [92].

Magnetic chitosan NP materials prepared to remove Hg(II), Cu(II), and Ni(II), were also examined for removal of Cu(II), Pb(II), and Cd(II) [93] and Ni(II) and Co(II) [94]. Table 2 summarizes research on NPs and chitosan magnetic NPs in the treatment of heavy metal ions. For instance, the preparation of chitosan NPs by ionic gelation was reported by [95,96]. Ali et al. (2018) utilized chitosan NPs for Fe(II) and Mn(II) removal with an adsorption capacity of 116.2 mg/g and 74.1 mg/g, respectively, with a dosage of 0.5–15 g/L [65]. Basaad et al. (2016) reported the adsorption of Fe(II) and Mn(II) with an effectiveness of 99.94% and 80.85%, respectively [97].

The preparation of magnetic modified chitosan using co-precipitation and cross-linking by glutaraldehyde is reported in the work of [98] that was used for the removal of Zn(II) [85], while magnetic chitosan NPs were used for the removal of As(V) and As(III) [98]. The initial Zn(II) concentration was taken as 1300 mg/L, and a magnetically modified chitosan dose of 300 g/L was applied, which removed 99% of the metal ions with the adsorption capacity of 32.16 mg/g, under these experimental conditions we have a high efficiency and a low adsorption capacity. The adsorption mechanism followed Langmuir-Freundlich isotherm means that we have a multilayer adsorption and not a specific adsorption [98]. Magnetic chitosan nanoparticles (MCNPs) have shown good results with a removal capacity of 95% (≈144.75 mg/g) for both As(V) and As(III) at a dose of 1.0 g/L and a concentration of 2 at nearly 11 mg/L within just 15 min and followed by Sips (Langmuir-Freundlich) isotherm [85]. In the work of Liu et al., the dominant mechanism, in this case, is the electrostatic attraction between the positive surface charges of the protonated chitosan amine functions and the negative charges of the arsenate ions. knowing that according to the authors, the MCNP is rich in –N of about 1.93 mmol/g (N/C ratio = 0.252) and the amine groups participate in the hydrogen bonding of the chitosan, and are known by their strong affinity against heavy metals.

**Table 2:** Chitosan-based nanomaterials for heavy metal removal.

**Biosorbent**	**Modification Method**	**Characterization**	**Metal**	**Ad. Efficiency (mg/g)**	**Models**	**Parameters**					**References**
						**pH**	**Time and Speed**	**Temperature**	**SD**	**C_0_ (mg/L)**	
Chitosan nanoparticles (CNPs)	Ionic gelation	FT-IR XRD TEM SEM	Fe(II) Mn(II)	116.2 74.1	Langmuir	2.5–7	120 min 180 tr/min	Room temperature	0.5–15 g/L	10–120	[65]
	Ionic gelation	FT-IR XRD SEM AFM TGA DSD	Cr (VI)	1395.32	Langmuir Freundlich Temkin	3–9	60min	Room temperature	1 g/100 mL	200	[96]
	Ionic gelation	TEM	Pb(II) Cd(II) Cr(V)	398 358 323.6	Langmuir	4	/	/	/	/	[99]
Magnetic CS—Gluta. (CMMC)	Co-precipitation and cross-linking	SEM TEM FTIR	Zn(II)	32.2	Langmuir Freundlich	5.0	30 min 100 rpm	25 °C	0.3 g/mL	50 and 1300	[98]
Magnetic chitosan particle (MCNPs)	Co-precipitation and cross-linking	UV	As(V) As(III)	65.5 60.2	Langmuir Freundlich	6.8	15 min 150 tr/min	Room temperature	1 g/L	0.2–50	[85]
Chitosan magnetic beads	Magnetite powder added to chitosan solution to form magnetic beads	SEM	Cd(II)	580	/	6.5	100 rpm	25 °C	0.15 g of dry chitosan	/	[100]
Thiourea-modified magnetic chitosan microsphere composite (TMCS)	Co-precipitation	XRD FTIR VSM Zeta Potential analyzer	Hg(II) Cu(II) Ni(II)	625.2 66.7 15.3	Langmuir Freundlich Temkin	5.0 5.0 5.0	8 h 150 rpm	28 °C	0.3 g of dry TMCS	/	[101]
**Biosorbent**	**Modification method**	**Characterization**	**Metal**	**Adsorption efficiency or capacity**	**Adsorption isotherm**	**Parameters**					**References**
						**pH**	**Stirring time and speed**	**Temp.**	**SD**	**C_0_ (mg/L)**	
CTS NPs	Ion gelation method	/	As(V)	90%	Sips	3.5	3 h 200 rpm	24 °C	0.025 g/50 mL	100	[102]
Magnetic chitosan nanoparticles (MCNPs)		DLS dynamic light scattering	Cd(II)	358 mg/g	Langmuir	4.6	12 h 300 tr/min	28 °C	1 g/L	0.5	[103]
Cross-linked chitosan magnetic modified with methionine-glutaraldehyde	New nano biosorbent based on cross-linking of chitosan and magnetic NPs (Chi-MG/Fe_3_O_4_)	XRD FTIR SEM	Cu(II) Pb(II) Cd(II)	172.4 mg/g 175.4 mg/g 163.9 mg/g	Langmuir	5.5	40 min 150 rpm	Room temperature	/	/	[93]
Composite chitosan—magnetite microparticles	Magnetic material was produced by oxidation of ferrous ions incorporated into a chitosan-Fe(II) complex	/	Ni(II) Co(II)	833.34 mg/g 588.24 mg/g	Langmuir	5.5	24 h 200 rpm	25 °C	0.1 g	340	[94]
Chitosan NPs	Ion gelation method using tripolyphosphate	FT-IR TEM	Fe(II) Mn)II) Zn(II) Cu(II)	99.94% 80.85% 90.49% 95.93%	/	7	30 min 100 tr/min	Room temperature	2 g/L	20 ppm	[97]
Magnetite Fe_3_O_4_ NPs coated with chitosan	Iron oxide NPs were synthesized using co-precipitation	TEM, FTIR XRD VSM	Cd(II) Cr(VI)	99% 98%	Langmuir Freundlich	6.5 2.5	10 min 400 rpm	22 °C	/	10, 20, 40 and 80 mg/L	[104]

Magnetic chitosan NPs Seyedi et al., (2013) and chitosan magnetic beads [100], used for Cd(II) removal, followed the Langmuir adsorption isotherm. Magnetic chitosan NPs, chitosan NPs, and Chi-MG/Fe_3_O_4_ have the adsorption capacity for cadmium removal of 580 mg/g, 358 mg/g, and 163.9 mg/g, respectively [93,99,100]. Sivakami et al. (2013) and Tang et al. (2007) reported the removal of hazardous Cr(VI). The initial concentration of Cr(VI) was 200 mg/L with an adsorbent dose of 10 g/L, kept for 60 min. With the optimum time of one hour, chitosan NPs prepared by ionic gelation removed 61–75% of the metal ions from an aqueous solution with an adsorption capacity of 1395.32 mg/g, which was considered to be very good [95,96]. Qi & Xu, (2004) prepared chitosan NPs by ionotropic gelation of chitosan and tripolyphosphatecross–linking to use for the removal of Pb(II) and it is used in the work of Sivakami et al. (2013) to eliminate the Cr(VI) to Cr(III) ions, the adsorption capacity was 398 mg/g and 323.6 mg/g for Pb(II) and Cr(V), respectively [64,96]. According to the authors [96], the high efficiency of materials against Cr(VI) is due to the electrostatic attraction between two oppositely charged ions, since the chromium(VI) ions in the solutions are present in the form of dichromate ions which are negatively charged and chitosan NPs having an overall positive surface charge. Zhou et al. (2009) reported Hg(II) removal with an adsorption capacity of 625.2 mg/g using chitosan composites designated as thiourea-modified magnetic chitosan microsphere composites (TMCS) [101], that is characterized by its high percentage of nitrogen (-N) and sulfur (-S). These composites showed and adsorption capacity of 66.7 mg/g and 15.3 mg/g for Cu(II) and Ni(II) respectively. The amine and sulfur groups present on the surface of TMCS are responsible for metal ion binding through chelation mechanisms. Amine and sulfur sites are the main reactive groups for metal ions though hydroxyl groups in the C-3 position [105,106]. Ample research has also been reported for the removal of heavy metals such as As(V) [103], Cu(II) [94,98], and Co(II) [95] as summarized in Table 2.

**Table 3:** Chitosan-based nanomaterials for dye removal.

Biosorbent	Modification Method	Characterization Method	Dyes	Adsorption Efficiency or Capacity	Adsorption Isotherm	Parameters					References
						pH	Stirring Time and Speed	Temperature	SD	C Initial (mg/L)	
Chitosan NPs	Ionic gelation	TEM	Rougeacide73 -Orange acide12 -Rouge acide18 -Noir acide26 -Direct bleu78	2.1 mmol/L 1.8 mmol/L 1.4 mmol/L 34.5 mg/g 52.6 mg/g	Langmuir	4	/	/	/	/	[99]
Glutaraldehyde cross-linked magnetic chitosan NPs	Glutaraldehyde-bonded tripolyphosphate	FT-IR TEM	FD&C blue1 D&C jaune5	475.6 mg/g 292.1 mg/g	Langmuir	3.0	60 min 150 tr/min	25 °C	50 mg/50 mL	50–1500	[86]
Magnetic β-cyclodextrin–chitosan/graphene oxide as nanoadsorbent	Co-precipitation method and chemical cross-linking	FTIR SEM TEM XRD	Methylene Bleu	84.3 mg/g	Langmuir Freundlich	11.0	180 rpm	25 °C	0.01 g and 100 mL	/	[107]
MCNCs	Glutaraldehyde cross-linking	/	Crystal violet Acid Red	333.3 mg/g (72%)	Langmuir Freundlich Temkin	7.0	140 min	25 °C	1.0 g	77	[108]
MCNCs	Reduction precipitation method	XRD	Acid Red	90.1 mg/g	Redlich–Peterson Langmuir Freundlich	3.0	/	/	1.0 g/L	/	[109]
Chitosan-coated magnetic mesoporous silica NPs	Chemical co-precipitation and chemical modification	FTIR XRD TEM TGA	Methylene Blue	43 mg/g	Langmuir Freundlich	7.0	90 min	25 °C	0.02 g	20	[110]
Magnetic chitosan NPs	Chemical co-precipitation	SEM, TEM FTIR, XRD VSM	RR-141 RY-14	99.5% (98.8 mg/g) 92.7% (89.7 mg/g)	Freundlich	4.0–10.00	30 min 120 rpm	25 °C	/	/	[111]

#### 4.1.2. Dye Removal

Dyes are chemical compounds that impart color when attached to the surface of fabrics, papers, and cosmetics. Dyes are used in the manufacture of ink, paint, and laboratories. The annual production of commercial dyes used in industry is estimated to be over 7 × 10^5^ tonnes per year. An average of 1 × 10^2^ tonnes per year is discharged into water bodies as waste. Such an amount of dye effluent in water can cause serious harm to all living things [45]. Prolonged exposure to toxic dyes leads to skin irritation, respiratory disorders, and even cancer [9]. For these reasons, researchers are interested in eliminating toxic molecules using materials such as NPs and magnetic NPs of chitosan, as shown by [99,112]. Table 3 shows some examples of chitosan NPs and experimental conditions for the elimination of toxic dyes. The main dyes used in laboratories, and textile and color industries are methylene blue, crystal violet, and acid red. Chitosan composites can successfully remove these dyes from wastewater along with other dyes such as rougeacide73, orange acide12, rouge acide18, noir acide26, and direct blue 78 [99].

Zhou et al. (2014) successfully prepared glutaraldehyde cross-linked magnetic chitosan NPs to remove FD&C blue1, and D&C jaune5 dyes with the adsorption capacity of 475.61 mg/g and 292.07 mg/g, respectively, within 1.0 h at a dosage of 1.0 g/L. The adsorption processes were spontaneous and exothermic, thus following the Langmuir isotherm, which suggested monolayer adsorption on the surface and confirmed low cytotoxicity [86].

Methylene blue was successfully adsorbed from an aqueous solution by various type of magnetic sorbents [107], including, magnetic β-cyclodextrin–chitosan/graphene oxide (as nano adsorbent). The study concluded that, with the least dose of 0.1 g/L, the composite of cyclodextrin-chitosan-graphene obtained an adsorption capacity of 84.3 mg/g, which is higher than that of chitosan-coated magnetic mesoporous silica NPs (43.03 mg/g) (Li et al. 2015) [85]. Magnetic chitosan nanocomposite and glutaraldehyde cross-linked magnetic chitosan nanocomposites were prepared for the removal of crystal violet and acid red, respectively [108,109]. Adsorption of crystal violet was possible with an adsorption efficiency of 72% and adsorption capacity of 333.3 mg/g at SD of 1g/L, C_0_ = 77 mg/L, and contact time of 140 min, while the adsorption capacity for acid red was 90.06 mg/g. The interaction between chitosan and CV is due to the binding between the –NH_2_ and –OH groups of chitosan and amino cationic groups of dye CV. Furthermore, according to the results of kinetics data obtained by researchers [108] the adsorption of CV on MC followed the intra-particle diffusion model, which implies CV adsorption on MCNC was a chemical process and electrostatic interaction, at the same time (a mixed mechanism).

#### 4.1.3. Uranium and Light/Heavy Rare Earth Metal Removal

Uranium belongs to the family of actinoid or actinide elements, which contains 15 consecutive chemical elements in the periodic table. These elements, especially uranium, are important due to the common property of radioactivity. The importance of uranium is its use in atomic weapons for their explosive power and in nuclear plants for the production of electrical power. Studies on the adsorption of traces of uranium U(VI) by using different nanomaterials such as magnetic chitosan NPs have increased research interest. Chitosan magnetic NPs are not only limited to heavy metal or dye removal but also extended to the removal of rare earth metals, Some studies are summarized in Table 4. In the study of Galhoum et al. (2015), materials based on magnetic chitosan NPs were prepared to extract La(III), Nd(III), and Yb(III) ions [84]. The authors reported La(III), Nd(III), and Yb(III) removal with adsorption capacities of 16.2 mg/g, 14.6 mg/g, and 12.9 mg/g, respectively, using cysteine-functionalized chitosan magnetic NPs.

There are limited studies on the removal of rare earth elements using chitosan composites. For instance, alanine-functionalized chitosan magnetic nano-based particles and serine-functionalized chitosan magnetic NPs were used by Galhoum et al. (2015d) for the scavenging of U(VI). The authors reported the successful removal of U(VI) with adsorption capacities of 85.3 mg/g and 116.5 mg/g of the prepared composites [73], respectively. Later, Galhoum et al. (2017) reported various NPs such as alanine-chitosan magnetic NPs, cysteine-chitosan magnetic NPs, serine-chitosan magnetic NPs, and ethylenediamine-tetraacetic acid- chitosan magnetic NPs for U(VI) removal [82]. It was observed that ethylenediamine-tetraacetic acid-chitosan magnetic NPs had the best results with an adsorption capacity of 185.0 mg of U(VI)/g, which was the same as diethylenetriamine-chitosan magnetic nano-based particles [113], Alanine-chitosan magnetic NPs had the least adsorption capacity (88.5 mg/g). The adsorption process onto composites followed the Langmuir isotherm model. Tetra-ethylenepentamine-modified chitosan magnetic resins obtained an adsorption capacity of 397 mg/g with an adsorbent dose of 0.1 g, an initial concentration of uranium of 6 mmol/L and an optimum time of 30 min [114].

**Table 4:** Chitosan-based nanomaterials for uranium and rare earth metals removal.

Biosorbent	Modification Method	Characterization Method	Metal	Adsorption Efficiency or/and Capacity	Adsorption Isotherm	Parameters					References
						pH	Stirring and Speed	Temperature	SD	C Initial	
Alanine-functionalized chitosan magnetic nano-based particles	Co-precipitation followed by hydrothermal treatment and after grafting	FTIR XDR TEM VSM	U(VI)	85.3 mg/g	Langmuir Dubinin-Radushkevich	3.6	50 min 200 rpm	25 °C	0.05 g and 50 mL	110 mg/L	[73]
Serine-functionalized chitosan magnetic nano-based particles		FTIR XDR TEM VSM	U(VI)	116.5 mg/g	Langmuir Dubinin-Radushkevich	3.6	50 min 200 rpm	25 °C	0.05 g and 50 mL	110 mg/L	[73]
EDTA-chitosan magnetic nanoparticles (CMNPs) Alanine-CMNPs Cysteine-CMNPs Serine-CMNPs	Grafting of –N functions by combined polymer precipitation and hydrothermal treatment	VSM XDR TEM	U(VI)	185 mg/g 88.5 mg/g 101.0 mg/g 120.5 mg/g 185 mg/g	Langmuir	4.0	45–60 min 200 rpm	25 °C	1 g/L	110 mg/L	[82]
TEPA modified chitosan magnetic resin	Cross-linking	SEM EDX	U(VI)	397 mg/g	Langmuir Freundlich Dubinin–Radushkevich	4.0	30 min 200 rpm	25 °C	0.1 g	6 mmol/L	[113]
DETA-chitosan magnetic nano-based particles	Co-precipitation method	FT-IR XRD AFM TEM VSM	U(VI)	185 mg/g	Langmuir Dubinin–Radushkevich (D–R)	4.0	40 min 200 rpm	25 °C	0.02 g	110 mg/L	[114]
Cysteine-functionalized chitosan magnetic nano-based particles	Co-precipitation and cross-linking	FTIR XRD TEM VSM	La(III) Nd(III) Yb(III)	16.2 mg/g 14.6 mg/g 12.9 mg/g	Langmuir	5.0	4 h 300 rpm	27 °C	0.05 g and 100 mL	100 mg/L	[84]
Alanine and serine functionalized magnetic nano-based particles	Co-precipitation	/	Nd(III) Yb(III)	9 mg/g 18 mg/g	Langmuir	5.0	4 h	/	/	/	[115]

### 4.2. Parameters Affecting the Chitosan Biosorbent Performance

There are various factors that affect the adsorption process, such as pH, temperature, contact time, initial concentration of pollutant, and the amount of adsorbent. These factors must be optimized to obtain maximum adsorption capacity.

The pH of a solution strongly affects the adsorption capacity of chitosan nanoparticles, such as increasing pH will increase adsorption. Magnetic chitosan nano-composites contain functional groups, such as –NH_2_, –NH, –COOH, –OH, and C=S. At higher H^+^ concentrations or lower pH, metal cations have to compete with H^+^ to be adsorbed onto the surface of magnetic chitosan nano-composites. However, beyond a certain pH range, a further increase leads to precipitation [45]. The pH also affects the surface of the adsorbent by charging the molecule itself or attached functional groups, which further enhances the attraction between the adsorbate and the adsorbent. Contact time is an important parameter in determining the maximum adsorption capacity at a fixed pollutant concentration; it is the minimum time required for a particular concentration of dye or metal to interact with the adsorbent [45]. After reaching T_eq_, the adsorption becomes constant or slow, which shows that all the adsorbent sites are occupied and that no vacant site is there to adsorb more pollutants. Among the advantages of using NPs in adsorption are the reduction in equilibrium time and the rapid adsorption rate. Generally, the equilibrium time of heavy metal adsorption onto magnetic chitosan nano-composites is lower than 1 h, except for certain types of metal and most dyes.

It is necessary to measure the adsorption capacity by varying the mass of the adsorbent. The percentage of removal of heavy metals and dyes increases with increasing doses of adsorbent. These increases are attributed to the increase in the number of active sites and the surface area of chitosan NPs. Sometimes, equilibrium is achieved if a certain adsorbent mass is exceeded. In other cases, a decrease in the adsorption capacity at an extra amount of adsorbent is observed. Researchers explain this phenomenon by aggregation of particles, which may also have a negative impact on the process because of the decrease in the surface area of the adsorbent. In practical applications, a minimum amount of adsorbent that is capable of satisfying the needs should be employed [116,117].

Similar to contact time and dosage, the initial pollutant concentration directly relates to the active sites during adsorption and helps determine the adsorption capacity of the adsorbent. Pollutant removal efficiency decreases with an increasing initial concentration of these pollutants because active sites become occupied and saturated over time. According to the results obtained by researchers, when the initial concentration of adsorbate increases, the adsorption first increases, and then at some point, it becomes constant, which shows that almost all the sites on the surface of the adsorbent are filled, and no vacant sites are available for adsorption [118,119,120].

Temperature is an important factor that affects the efficiency of adsorption. Studying the effect of temperature helps decide whether the process is exothermic or endothermic. When adsorption increases with increasing temperature, the process is an endothermic process. The increase in temperature increases the mobility of the pollutant, so the adsorption becomes faster due to the easy access of the adsorbate to the adsorbent. For example, Seyed Massoud Seyedi (2013) showed that the elimination of Cd(II) took place at 28 °C for chitosan and nanochitosan [103]. Furthermore, according to Table 4, researchers prefer to use chitosan NPs at an ambient temperature of 25 °C.

## 5. Predominant Mechanisms When Using Chitosan NPs

The adsorption mechanisms depend on the pH of the solution, pH_pzc_, chemical composition of chitosan and composite structure, chemical nature of the metal ions, nature of their charges (cations or anions) as well as the number of atoms that compose them (monoatomic or polyatomic) [121].

Knowing that the pH affects the surface of the adsorbent by charging the molecule itself or the functional groups attached, further enhances the attraction between the adsorbate and the adsorbent [45]. The adsorption of methyl orange (MO) on magnetic chitosan beads (MagCH) was tested in the work of L. Obeid et al., (2013) they noticed that the adsorption is strongly linked by the pH value of the medium The results indicated a better efficiency of 99% (≈779 mg/g) in the pH range of 3 to 5 due to electrostatic attraction between the negative functional groups of the dye and the positively charged magnetic beads [122].

In the work of Massoudinejad et al. (2019) [108], the pH_pzc_ of the Fe_3_O_4_ NPs was about 7.1, confirming the coating of chitosan, and it is evident that the MC nanocomposites were positively charged at pH < 6.5, which is consistent with the work of Chang and Chen (2005) who found the value of 6.7 [123]. While in the work of Zhou et al. (2009), the isoelectric point (IEP) of the TMCS was 5.92. This revealed that the TMCS microspheres were positively charged at pH < 5.92 and negatively charged at pH > 5.92. So, TMCS is positively charged with a pI of 5.92. Therefore, in acidic solutions, it is protonated and possesses electrostatic properties. Thus, it is also possible to adsorb metal ions through anion exchange mechanisms.

All types of nano-adsorbents have functional groups (active sites), such as carboxyl, hydroxyl, and amine groups, which bind to heavy metals either by ion exchange (where –COOH groups are involved) or by a complexation mechanism (where –COOH groups, –OH, –SH, and –NH_2_ may be involved). Metal ions generally bind to the magnetic chitosan nano-composites via the available functional groups, and the mechanism is proposed according to the active sites available on the surface of these materials. Each site gives several possibilities of interaction; therefore, the mechanism can be considered by the researcher, taking into consideration the nature of the site and the pollutant.

The binding mechanisms are influenced by both the type of ion/dyes and the functional groups of the active sites. The amine groups (–NH_2_) were the most reactive and mainly responsible for fixing metals rather than the hydroxyl groups carried by the C-3 carbon [123]. The strong attraction of the free electron pair to the nitrogen atom was greater than that of the oxygen atom, and it contributed to the formation of a metal complex by sharing its free electron pair with a metal ion [124]. In particular, the existence of the functional groups containing oxygen, such as carboxylic, phenolic, or lactone functions, results in an acidic character, while the presence of pyrones, chromene-type functions induces a basic character [125]. Depending on their nature and concentration, these surface functional groups can influence an adsorbent’s adsorption capacity and the hydrophilic/hydrophobic character.

The three main mechanisms for fixing pollutants on chitosan mentioned in the literature are (i) complexation and/or chelation, (ii) ion-exchange/electrostatic interaction with anions, and (iii) formation of ternary complexes. Adsorption mechanisms are much more complex with magnetic NPs than with normal chitosan.

There are several models for modeling and analyzing the experimental data, such as the Freundlich, Langmuir, Sips, and Dubinin-Radushkevich isotherm models. According to the best-adapted model of isotherms and kinetics, we can understand the sorption mechanisms and can give an idea that is not confirmed on the nature/type of adsorption, so the successful fitting of the kinetic or isotherm model alone does not validate any evidence about chemisorption or physisorption.

For example, the Dubinin-Radushkevich model discriminates between physical and chemical mechanisms [83]. The application of the Sips or/and Langmuir model is linked directly to the nature of the surface, the homogeneity or heterogeneity of the surface (identical/or different active site), monolayer (in the case of chemical and specific adsorption, which must be carried out in a specific active site) or multilayer (in the case of physical adsorption) or also mixed sorption according to the results obtained. Despite the complexity of this mechanism, we can say that chemisorption occurs via the hydroxyl and/or amine groups of chitosan or additional functional groups present (thiol, amino, carboxyl, etc.).

## 6. Conclusions and Future Perspectives

A wide range of treatment technologies is developing daily for water purification to meet the current demands. Advances in nanoscale science and engineering offer new opportunities to develop more cost-effective and environmentally acceptable water treatment technology. Nano means reducing the size of any material to the nanoscale, and in doing so, the properties of that material change dramatically. Recent research has indicated that chitosan nanomaterials, due to their unique physicochemical properties, structure, and surface characteristics, are useful tools for the removal of organic and inorganic pollutants. These materials can remove metal ions at low concentrations, with high selectivity and adsorption capacity. Nowadays, nanotechnology has become an advanced technology that gives scientists a tool to prepare effective adsorbents to remove contaminants from water. Nanoscale adsorbents have high performance due to their large specific surface area and quantum size effect, which could cause them to exhibit higher capacities for pollutants, particularly metal ions. We conclude that there are different methods for the development of chitosan NPs. Among these methods, covalent cross-linking, electrostatic interactions, and microemulsion methods are widely accepted. At the same time, there are different methods of analysis to confirm the success of the chemical modification, such as FT-IR, XRD, SEM, TEM, AFM, DLS, and VSM.

In addition, the use of NPs and magnetic NPs of chitosan offers several advantages in the field of adsorption compared to the initial powder, because chitosan is already known for its great affinity with respect to most pollutants and this form gives more advantages and reinforces its stability and efficiency when used in the field of wastewater treatment (rapid separation, rapid kinetics, stability, easy and total desorption, large specific surface).

Further improvements should be made in the direction of developing materials with greater stability and the ability to simultaneously remove multiple contaminants under complex conditions. In addition, there is a need to synthesize inexpensive, efficient, and recyclable adsorbents for their practical applications. These techniques constitute reproducible processes with excellent size control, but the use of a chemical cross-linker, such as glutaraldehyde, and some organic solvents, can be detrimental to the final biocompatibility of the NPs. This review is a compilation of recent chitosan-nanomaterial composites reported recently for the removal of heavy metals, dyes, rare earth metals, or radioactive materials. Comparison of different composites, their preparation method, and their working conditions are summarized in this paper, which allows the reader to apply the modifications to previously available methods. Chitosan is a wonder material, which can be utilized in different forms. One can take the opportunity to read this review and further research the unlocked potential of this marine waste-extracted chitosan. In the present era of fast development, where environmental pollution is a huge global problem, chitosan gives us the opportunity to prepare a plethora of composites for different applications.

## Figures and Tables

**Table 1:** Methods commonly used to characterize chitosan NPs.

Transmission Electron Microscopy (TEM)	Fourier-Transform Infrared Spectroscopy (FTIR)	X-ray Diffraction (XRD)
used to examine the size, shape, and morphology of the NPs	used to detect or identify the functions of chitosan NPs, and to confirm the presence of iron in the structure	used to examine the crystallinity of chitosan and chitosan NPs
Atomic force microscopy (AFM)	Dynamic light scattering device (DLS)	Scanning electron microscopy and transmission electron microscopySEM and SEM-EDX
used to visualize the rough nature of chitosan NPs, which facilitate the adsorption of heavy metals	used to determine the size of NPs. To confirm the synthesis of magnetic NPs, it is necessary to use a vibrating sample magnetometer, which characterizes the magnetic properties of chitosan.	analyses the shape and type of surface used to determine the morphology of chitosan NPs

## Data Availability

Not applicable.
